# Brain functional specialization and cooperation in Alzheimer's disease

**DOI:** 10.1002/brb3.3550

**Published:** 2024-06-06

**Authors:** Yue Wu, Manman Gao, Lingling Lv, Yibing Yan, Liying Gao, Zhi Geng, Shanshan Zhou, Wanqiu Zhu, Yongqiang Yu, Yanghua Tian, Gong‐Jun Ji, Panpan Hu, Xingqi Wu, Kai Wang

**Affiliations:** ^1^ Department of Neurology the First Affiliated Hospital of Anhui Medical University Hefei Anhui Province China; ^2^ Anhui Province Key Laboratory of Cognition and Neuropsychiatric Disorders Hefei Anhui Province China; ^3^ Department of Psychology and Sleep Medicine the Second Hospital of Anhui Medical University Hefei Anhui Province China; ^4^ Collaborative Innovation Center of Neuropsychiatric Disorders and Mental Health Hefei Anhui Province China; ^5^ Department of Radiology the First Affiliated Hospital of Anhui Medical University Hefei Anhui Province China; ^6^ Institute of Artificial Intelligence Hefei Comprehensive National Science Center Hefei Anhui Province China; ^7^ The School of Mental Health and Psychological Sciences Anhui Medical University Hefei Anhui Province China

**Keywords:** Alzheimer's disease, cerebral specialization, interhemispheric cooperation, resting‐state functional magnetic resonance imaging

## Abstract

**Background:**

Cerebral specialization and interhemispheric cooperation are two vital features of the human brain. Their dysfunction may be associated with disease progression in patients with Alzheimer's disease (AD), which is featured as progressive cognitive degeneration and asymmetric neuropathology.

**Objective:**

This study aimed to examine and define two inherent properties of hemispheric function in patients with AD by utilizing resting‐state functional magnetic resonance imaging (rs‐fMRI).

**Methods:**

Sixty‐four clinically diagnosed AD patients and 52 age‐ and sex‐matched cognitively normal subjects were recruited and underwent MRI and clinical evaluation. We calculated and compared brain specialization (autonomy index, AI) and interhemispheric cooperation (connectivity between functionally homotopic voxels, CFH).

**Results:**

In comparison to healthy controls, patients with AD exhibited enhanced AI in the left middle occipital gyrus. This increase in specialization can be attributed to reduced functional connectivity in the contralateral region, such as the right temporal lobe. The CFH of the bilateral precuneus and prefrontal areas was significantly decreased in AD patients compared to controls. Imaging‐cognitive correlation analysis indicated that the CFH of the right prefrontal cortex was marginally positively related to the Montreal Cognitive Assessment score in patients and the Auditory Verbal Learning Test score. Moreover, taking abnormal AI and CFH values as features, support vector machine‐based classification achieved good accuracy, sensitivity, specificity, and area under the curve by leave‐one‐out cross‐validation.

**Conclusion:**

This study suggests that individuals with AD have abnormal cerebral specialization and interhemispheric cooperation. This provides new insights for further elucidation of the pathological mechanisms of AD.

## Graphical abstract

Dysfunction of specialization(AI)and interhemispheric cooperation (CFH) was observed in our patients, which may comprise a potential neural substrate for cognitive impairment during AD progression.

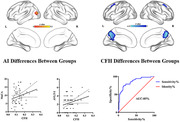

## 1 INTRODUCTION

Two vital organizing principles for normal functions of the brain are specificity and coordination between hemispheres (Gazzaniga, 2000). They are reinforcing and inseparable. Specialization and cooperation are foundation of the brain information processing and transmission (Karolis et al., 2019). Typically, the left hemisphere (LH) is more specialized for language (Geschwind & Levitsky, 1968) and the right hemisphere (RH) is more specialized for function, such as attention (Spagna et al., 2020). Cognitive processes can be organized more efficiently owing to the cerebral specialization (Rogers et al., 2004). In addition, hemisphere cooperation is essential for the advanced cognition, such as, memory. Encoding verbal information activates the LH (Golby et al., 2001) whereas memory encoding some nonverbal information (unfamiliar faces or abstract patterns) activates the RH (Kelley et al., 1998). The bilateral cerebral areas are activated when namable objects are encoded (Kelley et al., 1998). Taken these together, the bilateral hemispheres process information not only separately, but also jointly. However, specificity and coordination become dysfunctional in neurodegeneration diseases (Minkova et al., 2017).

Alzheimer's disease (AD) is characterized by gradual and progressive cognitive decline, which is the most prevalent cause of dementia (Arvanitakis et al., 2019). AD is typically associated with the accumulation of amyloid‐beta and tau pathologies in neurofibrillary tangles, leading to cognitive deterioration (Jack et al., 2018; Malpetti et al., 2020). The uneven distribution of tau pathologies may indicate the involvement of brain functional specialization or impaired collaboration in the development of AD (Frings et al., 2015; Janota & Mountjoy, 1988).

Molecular biology and neuroimaging studies have provided evidence of atypical cerebral specialization in individuals with AD. Single nucleotide polymorphisms (SNPs) in genes are associated with structural shape asymmetries (Wachinger et al., 2018). Abnormal asymmetric metabolism, blood flow, and neuropathology are observed in molecular neuroimaging studies (Whitwell et al., 2018). Dysfunctional hemispheric specialization in AD patients has been demonstrated by resting‐state functional magnetic resonance imaging (rs‐fMRI) (Wu et al., 2020). Notably, previous studies were based on altered structural lateralization, which may not be sensitive at the function and voxel level. To avoid the potential bias of anatomical asymmetry, a novel connectome‐based index, namely, the autonomy index (AI), is proposed. In the field of functional specialization, AI has become a pivotal method of measurement, demonstrating its capability to operate at a granular level, namely, the voxel level. Compared to previous functional lateralization methods, this index does not rely on structurally symmetrical regions. AI has emerged as a dependable metric for quantifying the functional specialization observed in both healthy individuals and patients (Mueller et al., 2015; Wang et al., 2014).

Interhemispheric collaboration is essential for integrating information from both hemispheres of the brain. It plays a crucial role in complex cognitive processes such as semantic and sensory processing, attention modulation, and working memory (Davis & Cabeza, 2015). Neurodegenerative diseases often disrupt this coordination, leading to impaired cognitive functions (Li et al., 2018). Functional magnetic resonance imaging (fMRI) studies can measure interhemispheric cooperation by examining the functional connectivity (FC) between homotopic regions. Homotopic regions refer to the corresponding regions in each hemisphere, which can be identified by normalizing an individual's brain to a symmetry atlas (Zuo et al., 2010). However, it is important to consider that the bilateral hemispheres of the brain are not perfectly symmetrical (Luders et al., 2004). This asymmetry may introduce unexpected bias in the results. A more reliable approach is to define homotopic regions based on functional characteristics rather than solely on structural features. By doing so, we can obtain more precise cross‐hemispheric cortical maps and better understand the functional correspondences between homotopic regions (Jo et al., 2012).

In this study, our objective was to investigate the two inherent architectures of brain function, namely specificity and coordination, in individuals with AD. To achieve this, we employed artificial intelligence techniques to estimate cerebral specialization. Additionally, we devised a new measure, termed the Connectivity between functionally homotopic voxels (CFH), to assess interhemispheric cooperation. The CFH index is based on the connectivity between functionally homotopic voxels, wherein the functional homotopic region of a specific voxel is identified as the location showing the highest FC value in the opposite hemisphere. Higher CFH values indicate greater communication between the hemispheres (Sun et al., 2022). We compared AI and CFH between AD patients and sex‐ and age‐matched healthy controls (HCs). AI and CFH abnormalities were observed and the relationship between clinical measures and abnormal AI or CFH was determined. Our hypothesis included the following points: (1) patients with AD would exhibit dysfunction in AI and CFH; (2) abnormal AI or CFH was associated with decreased cognitive test scores; (3) the presence of abnormal AI and CFH could serve as reliable indicators for distinguishing between patients with AD and HCs.

## 2 METHODS

### 2.1 Participants

We enrolled a total of 64 patients diagnosed with AD from the First Affiliated Hospital of Anhui Medical University in China between September 2017 and May 2021. The diagnosis of AD was made by a specialist following the criteria established by the National Institute of Neurological and Communicative Disorders and Stroke and the AD and Related Disorders Association (NINCDS‐ADRDA) (McKhann et al., 1984). Specifically, the inclusion criteria were as follows: (a) meeting the criteria for possible or probable AD; (b) having a Mini‐Mental State Examination (MMSE) score less than 27; and (c) having Clinical Dementia Rating (CDR) scores ranging from 0.5 to 2. Patients with substance use disorders, other neurological diseases, and life‐threatening somatic diseases were excluded from the study. We also included 52 healthy control (HC) individuals who were either recruited from the local community through advertisements or were spouses of the study participants. The HC group met the following criteria: normal cognitive function, no history of neurological or psychiatric disorders, no use of psychoactive medications, an MMSE score equal to or greater than 27, and a CDR score of 0.

All participants included in this study were right‐handed individuals who gave their written consent after being fully informed about the research purposes and procedures. The study was conducted in compliance with the latest revision of the Declaration of Helsinki. The experimental protocols were approved by the ethics committee of the Anhui Medical University.

### 2.2 Neuropsychological assessment

We administered a series of clinical and neuropsychological assessments to all participants. The purpose was to determine a clinical diagnosis. The specific tests we conducted were as follows: (i) We used the MMSE test and Montreal Cognitive Assessment–Beijing Version (MoCA) to evaluate overall cognitive function. (ii) The Clinical Dementia Rating (CDR) was used as an indicator of disease severity. (iii) The Lawton–Brody Activities of Daily Living (ADL) scale was employed to assess daily functioning abilities. (iv) We utilized the Hamilton Anxiety Scale and Hamilton Depression Rating Scale to measure affective symptoms. (v) Memory evaluation was conducted using the Auditory Verbal Learning Test (AVLT), which consisted of AVLT—immediate (AVLT‐I), AVLT—delay (AVLT‐D), and AVLT—recognition (AVLT‐R). (vi) Visual‐spatial and executive abilities were assessed through the clock drawing test (CDT). (vii) Language skills were evaluated using the verbal fluency test‐animal (VFT). (viii) Attention was assessed through the digital span forward (DS‐F) and digital span backward (DS‐B) tests. In summary, these assessments provided a comprehensive evaluation of the participants' clinical and neuropsychological profiles.

### 2.3 MRI data acquisition

We conducted structural and functional MRI scans on each participant using a 3T scanner (Signa HDxt 3.0T, General Electric HD 750 w, Buckinghamshire, UK) at our institution. During the rs‐fMRI scans, participants were instructed to close their eyes and to avoid falling asleep or thinking about anything specific. For the structural MRI, we obtained high‐resolution sagittal three‐dimensional T1‐weighted images using a brain volume sequence. The imaging parameters were as follows: repetition time of 8.676 ms, echo time ratio of 3.184 ms, flip angle of 8°, field of view of 256 × 256 mm^2^, matrix size of 256 × 256, slice thickness of 1 mm, voxel size of 1 × 1 × 1 mm^3^, and a total of 188 sections. As for the rs‐fMRI, we used a standard echo planar imaging sequence. The imaging parameters were as follows: repetition time of 2000 ms, echo time ratio of 22.5 ms, flip angle of 30°, matrix size of 64 × 64, field of view of 220 × 220 mm^2^, slice thickness of 4.0 mm, and 33 continuous slices with a voxel size of 3.4 × 3.4 × 4.6 mm^3^. The duration of the resting‐state fMRI sequence was 6 min and 10 s.

### 2.4 MRI preprocess

We used the Advanced Data Processing Assistant for Resting‐State Functional MR Imaging toolkit, a component of the Data Processing & Analysis for (Resting‐State) Brain Imaging software (DPABI) (Yan et al., 2016), along with the Resting‐State Functional MR Imaging Toolkit (REST; http://www.restfmri.net) and the statistical parametric mapping software package (SPM12; www.fil.ion.ucl.ac.uk/spm) to acquire the rs‐fMRI data. To ensure data quality, we discarded the initial 10 volumes to avoid any unsteady state. After that, we conducted slice‐timing correction and realignment. Each individual functional image was then coregistered to its respective structural image. We performed spatial normalization using unified segmentation of the structural images. To address potential confounding factors, we included nuisance regressors such as the 24 Friston motion parameters, white matter high signal, cerebrospinal fluid signal, and linear trends as regressors to account for drifts in the BOLD signal. Furthermore, we applied temporal bandpass filtering (0.01−0.1 Hz) after the nuisance regression. Finally, we conducted motion scrubbing to remove time points with high motion.

### 2.5 AI calculation

For there were different number voxels between each hemisphere, total numbers of voxels in each the hemisphere should be considered. The equation used for calculating the AI within the entire brain is as follows:

AI=Ni/Hi−Nc/Hc.

In the ipsilateral and contralateral hemispheres, Ni and Nc represent the counts of voxels that showed significant correlation (*r* > .25, *p* < .001) with each voxel. Hi indicates the total voxel count in the ipsilateral hemisphere, while Hc represents the total voxel count in the contralateral hemisphere. Finally, we generated an AI map for each participant and used it in subsequent analyses. An 8‐mm full width at half‐maximum Gaussian kernel was used for the individual AI map smoothing.

### 2.6 CFH computation

To overcome the limitations of traditional connectivity analysis based on structural symmetry between regions, we computed the connectivity between functionally homologous regions. In simple terms, we took two steps: (1) Defining homologous regions. For a given voxel, we performed seed‐based whole‐brain FC analysis and averaged the FC across all participants. In the contralateral hemisphere, the voxel with the highest connectivity value was defined as the seed region for homology. (2) Computing homologous connectivity maps. The CFH value of each voxel was defined as the Pearson correlation coefficient with the contralateral seed region. Finally, we obtained CFH maps for each participant and used them for further analysis. Individual CFH maps were smoothed using an 8 mm full width at half‐maximum Gaussian kernel. In our previous study, we employed AI and CFH measures to investigate Parkinson's disease (Sun et al., 2022).

### 2.7 AI and CFH analysis

We conducted a comparative analysis of the AI and CFH maps between the AD and HC groups. Independent samples *t*‐tests were conducted using the DPABI software within a gray‐matter mask, excluding the cerebellum. All statistical maps were corrected with a Gaussian random field (GRF) method with the significance of voxel level set at *p* < .0001, and that of cluster level set at *p* < .05, to control for type I error. To analyze the differences between AI and CFH in more detail, we extracted the instances where there were significant differences between the two. These instances were then centered around the peak point of the cluster, with a radius of 3.5 mm. This approach allows for a focused analysis of the variations between AI and CFH.

### 2.8 Statistical analyses

The clinical and demographic data were analyzed using IBM SPSS Statistics 20.0 software (IBM Corp., Armonk, NY, USA). Parametric data were expressed as means and standard deviations and analyzed using two‐sample *t*‐tests for the neuropsychological assessments. Nonparametric data were presented as medians and interquartile ranges and analyzed using the Mann–Whitney *U* test. Moreover, we performed a correlation analysis between FC and neuropsychological assessments to investigate the relationship between neuroimaging measures and cognitive impairment. Statistical significance was defined as *p* < .05.

### 2.9 Applying a support vector machine (SVM) technique for analyzing patterns in AI and CFH

In order to evaluate whether the identified neural metrics can be used as imaging biomarkers to distinguish between patients with AD and HCs, we used a linear support vector machine (SVM) approach within the LIBSVMs toolkit in MATLAB for classification using the linear kernel setting (Chang & Lin, 2011). The features used for classification were AI and CFH, which showed significant differences between the two groups. We employed a leave‐one‐out cross‐validation (LOOCV) strategy, where in each fold, one participant was left out and used as the testing set, while the remaining participants were used as the training set. This process was repeated for each participant with a total number of folds equal to the total number of participants. The training and test sets were labeled as AD or HC. Using the SVM procedure, a predicted label was obtained for each fold. By comparing the true and predicted labels, we obtained the classification accuracy, sensitivity, specificity, and area under the receiver operating characteristic curve (AUC). The performance of the classifier was evaluated based on the results of the cross‐validation. The significance of the accuracy was determined using a permutation test involving 5000 permutations. In the permutation test, the labels of the individuals were randomly shuffled, and the LOOCV strategy was applied based on the new labeling. This process yielded a new classification accuracy for each permutation. Based on the distribution of these 5000 accuracy values, we can infer the significance of accuracy in the original labeling condition. The statistical significance was set at *p* < .05.

## 3 RESULTS

### 3.1 Characteristics related to demographics and clinical aspects

The AD and HC groups showed a similar distribution in terms of age and sex, with no significant differences observed, but differences in years of education (*p* < .001) between the two groups. However, it is worth noting that there were significant differences in various cognitive assessment scores between the two groups. The AD group demonstrated significantly worse performance in MMSE (*p* < .001), MoCA (*p* < .001), CDR (*p* < .001), ADL (*p* < .001), AVLT‐I (*p* < .001), AVLT‐D (*p* < .001), AVLT‐R (*p* < .001), DS‐F (*p* < .001), DS‐B (*p* < .001), CDT (*p* < .001), and VFT (*p* < .001) scores (Table 1).

**TABLE 1:** Demographic data and neuropsychological assessment.

Variable	HC	AD	*t/Z/χ* ^2^	*p*
Demographic				
Age	63.43 (9.21)^a^	64.94 (8.52)^a^	0.90	.368^c^
Gender (M/F)	21/31	25/39	0.02	.885^d^
Education	10.00 (6.00)^b^	6.00 (9.00)^b^	4.19	<.001^e^
Neuropsychological assessment				
MMSE	29.00 (3.00)^b^	15.48 (6.02)^a^	8.99	<.001^e^
MoCA	25.00 (6.00)^b^	9.40 (5.05)^a^	8.72	<.001^e^
CDR	0.00 (0.00)^b^	1.00 (1.00)^b^	9.46	<.001^e^
ADL	20.00 (0.00)^b^	28.00(10.00)^a^	7.72	<.001^e^
HAMA	4.00 (5.00)^b^	5.00(7.00)^a^	0.63	.530^e^
HDRS	2.00 (5.00)^b^	5.00 (5.00)^a^	2.50	.012^e^
ALVT‐I	8.29 (1.85)^a^	2.42 (1.91)^a^	16.53	<.001^c^
ALVT‐D	9.00 (4.25)^b^	0.00 (1.00)^b^	8.92	<.001^e^
ALVT‐R	14.00 (1.00)^b^	10.50 (6.00)^b^	6.58	<.001^e^
DS‐forward	7.00(3.00)^b^	5.00 (2.00)^b^	4.94	<.001^e^
DS‐backward	4.00 (2.00)^b^	3.00 (1.00)^b^	5.32	<.001^e^
CDT	4.00 (1.00)^b^	1.00 (1.00)^b^	6.60	<.001^e^
VFT	17.98 (4.08)^a^	8.17 (4.62)^a^	11.88	<.001^c^

^a^ Parametric variables.

^b^ Nonparametric variables.

^c^ Two sample *t*‐test.

^d^ Chi‐square test.

^e^ Mann–Whitney *U* test.

Abbreviations: ADL, Activities of Daily Living; AVLT‐D, Auditory Verbal Learning Test—delay; AVLT‐I, Auditory Verbal Learning Test—immediate; AVLT‐R, Auditory Verbal Learning Test—recognition; CDR, Clinical Dementia Rating; CDT, The Clock Drawing Test; DS‐B, Digital Span Backward; DS‐F, Digital Span Forward; HAMA, the Hamilton Anxiety Scale; HDRS, Hamilton Depression Rating Scale; MMSE, Mini‐Mental State Examination; MoCA, Montreal Cognitive Assessment—Beijing Version; VFT, Verbal Fluency Test.

### 3.2 Cerebral specialization

**FIGURE 1:**
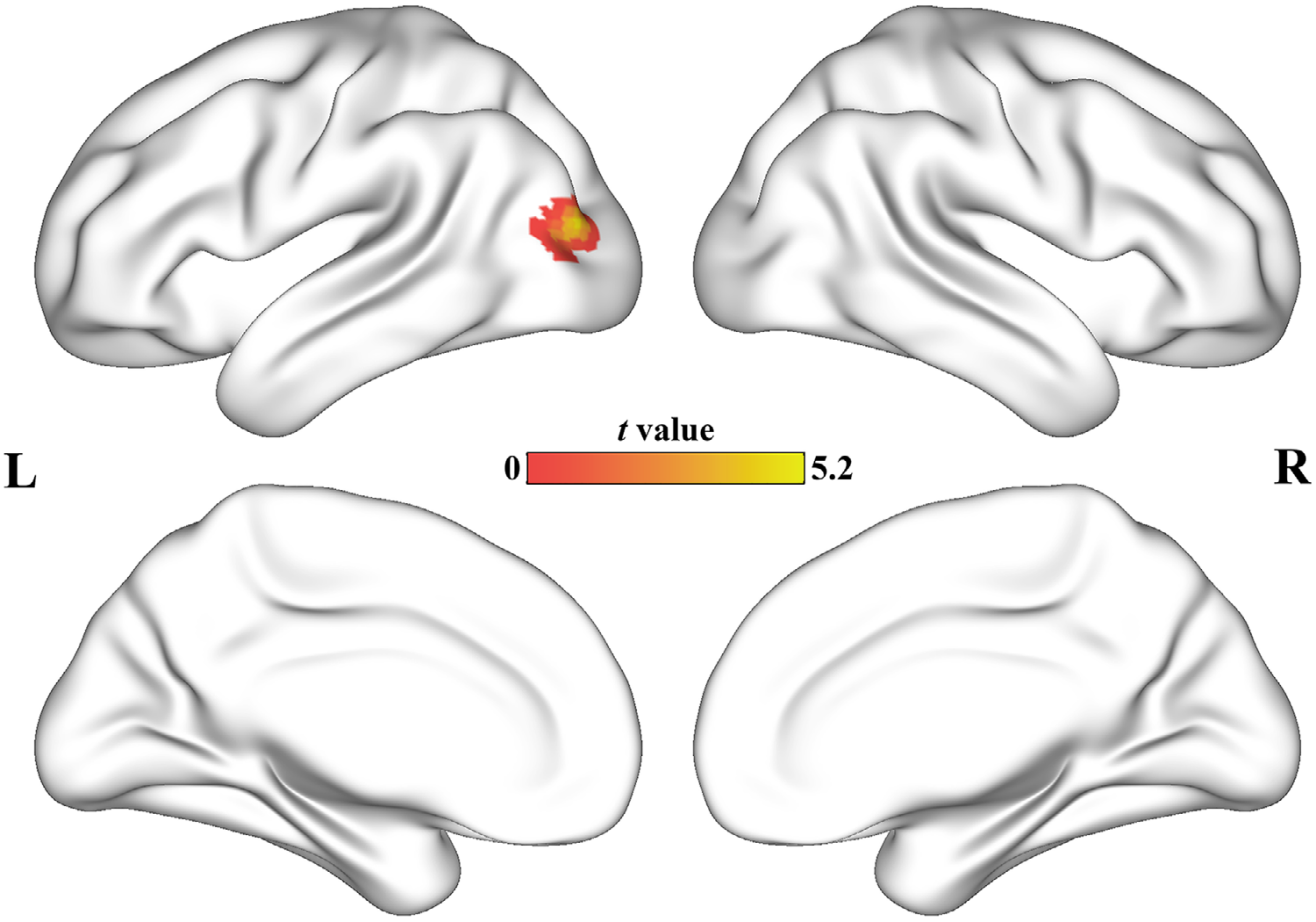
AI differences between groups. AI of the left middle occipital lobe was increased in AD group.

Patients diagnosed with AD exhibited a significantly stronger activation in the left middle occipital lobe (MOL) compared to the control group (peak *t*‐value = 5.27, MNI coordinates = [−39, −84, 15], cluster size = 47 voxels). To gain a more detailed understanding of the changes in connectivity of the left MOL, we conducted a comprehensive whole‐brain FC analysis between the two groups. The seeds for the FC analysis were defined as the cluster located in the left MOL. Subsequently, the resulting correlation coefficients were transformed into z‐scores using Fisher's *z*‐transformation. It is noteworthy that our approach for statistical analysis was consistent with the method described in Section 2.7. Patients with AD show decreased FC in the right temporal gyrus (MTG). Figure 1 shows the results of the AI analyses. The scatter diagram of AI is shown in the Figure S1. The FC difference between MOL and MTG is shown in the Figure S2 and Table S1 in supplementary materials.

### 3.3 Interhemispheric cooperation

**FIGURE 2:**
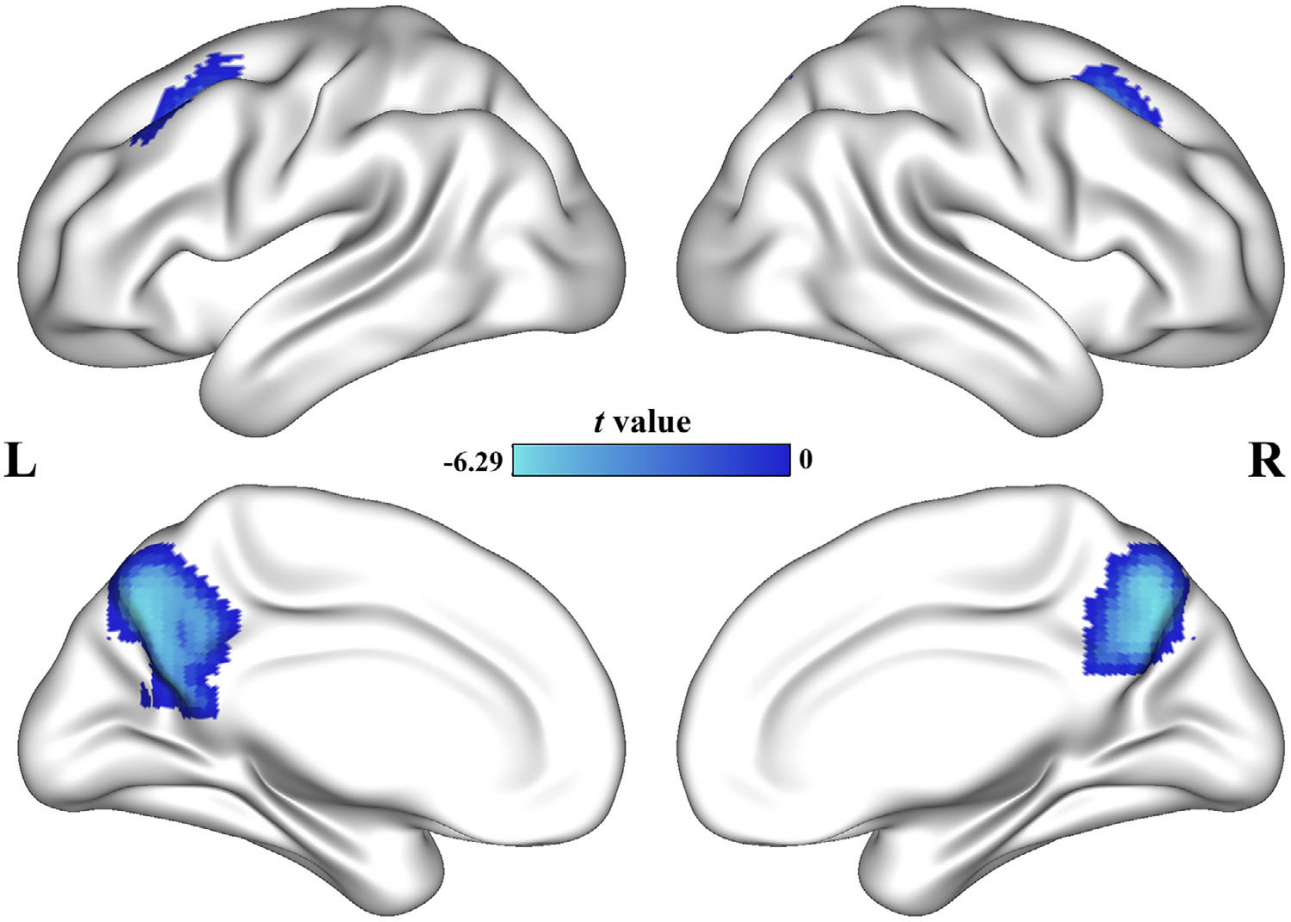
CFH differences between groups. CFH of bilateral precuneus and prefrontal cortex was decreased in AD group.

The AD group showed decreased CFH in the bilateral precuneus (left: peak *t*‐value = 5.92, MNI coordinates = [−9, −69, 42], cluster size = 274 voxels; right: peak *t*‐value = 6.21, MNI coordinates = [6, −69, 39], cluster size = 196 voxels) and bilateral prefrontal cortex (left: peak *t*‐value = 5.66, MNI coordinates = [−27, 15, 45], cluster size = 78 voxels; right: peak *t*‐value = 4.90, MNI coordinates = [27, 21, 45], cluster size = 40 voxels). Figure 2 shows the results of the CFH analyses. Table 2 shows the detail results of the AI and CFH. The scatter diagram of CFH is shown in Figure S3.

**TABLE 2:** Brain regions of AI or CFH differences between groups.

		Peak MNI coordinates					
Metrics	Brain regions	*x*	*y*	*z*	Voxels	*t*	*p _GRF_*_‐corr_
AI							
	L middle frontal cortex	–39	–84	15	47	5.27	<.05
CFH							
	L precuneus	–9	–69	42	274	5.92	<.05
	R precuneus	6	–69	39	196	6.21	<.05
	L prefrontal cortex	–27	15	45	78	5.66	<.05
	R prefrontal cortex	27	21	45	40	4.90	<.05

Abbreviations: AI, autonomy index; CFH, connectivity between functionally homotopic voxels; L, left; R, right.

### 3.4 Correlation analyses

**FIGURE 3:**
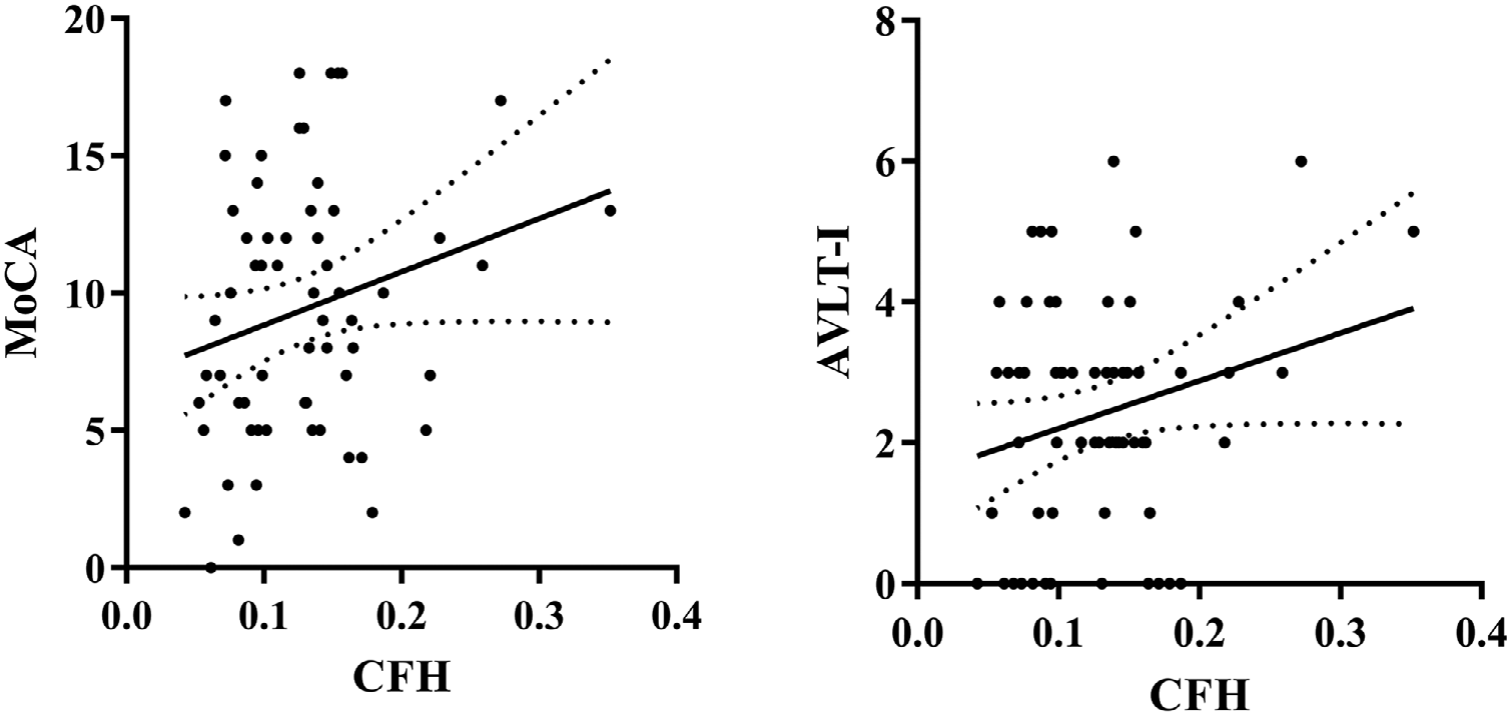
Results of the correlation analysis. Left panel: The CFH of the right prefrontal cortex was marginally positively correlated with the MoCA scores (*r *= .24, *p *= .066) in the AD group. Right panel: The CFH of the right prefrontal cortex was marginally positively correlated with the AVLT‐immediate scores (*r *= .24, *p *= .058) in the AD group.

Considering the influence of years of education on cognition, it was used as a covariate for correlation analysis. In the AD group, we observed a slight positive correlation between the right prefrontal cortex's CFH and MoCA scores (*r* = .24, *p* = .066), as well as between the CFH of the same brain region and AVLT‐immediate scores (*r* = .24, *p* = .058). However, we did not find any significant correlation between AI and other clinical characteristics of AD. To visualize these findings, please refer to Figure 3, which displays the results of the correlation analysis.

### 3.5 Classification results

**FIGURE 4:**
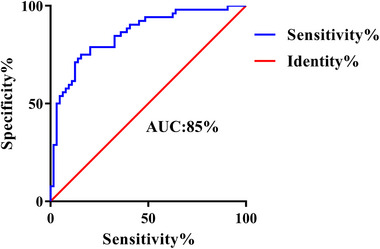
The SVM classification results. The linear SVM classifier achieved accuracy of 79.3%, sensitivity of 75.0%, specificity of 82.8%, and AUC of 85%.

By employing AI and CFH values to identify variations in brain regions between groups, we employed a linear SVM classifier. This classifier achieved an accuracy of 79.3%, a sensitivity of 75.0%, a specificity of 82.8%, and an AUC of 85%. The significance of the SVM was confirmed through a permutation test (*p* < .001). For a visual representation of the classification results, please refer to Figure 4.

## 4 DISCUSSION

This study aimed to investigate cerebral specialization and interhemispheric cooperation in patients with AD using two novel methods, AI and CFH, computed from rs‐fMRI data. First, we found that patients with AD exhibited abnormally increased specialization of the left MOL which resulted from a decreased contralateral FC (i.e., the right MTG). Additionally, our investigation revealed a disruption in the coordinated activity between the bilateral precuneus and prefrontal regions in AD patients. These regions exhibited impaired cooperation with their corresponding areas in terms of function. Moreover, we found a correlation between abnormalities in the CFH (coordinated functional hubs) and cognitive assessment scores in individuals with AD. In addition, the differences in AI and CFH could be regarded as features for classifying the two groups. The findings mentioned above provide valuable insights into the fundamental changes in the brain's functional organization in AD. These alterations are crucial for enhancing our understanding of the underlying mechanisms driving the development and progression of this condition.

Cerebral specialization plays a fundamental role in the intricate functioning of the human brain. Both neuropathology and imaging have shown asymmetric hemisphere dysfunction in AD (Ge et al., 2021; Roe et al., 2021; Walker et al., 2021). Previous studies showed that the leftward structural lateralization in the AD progression (Madsen et al., 2010; Wu et al., 2020). Our results demonstrate the integration of structural and functional lateralization abnormalities in AD. Abnormal specialization was observed in the left middle occipital gyrus. The occipital lobe is a vital area for memory encoding (Golby et al., 2005) and deactivation of the occipital lobe is a risk factor for AD (McDonough et al., 2020). Our results indicate a dysfunctional occipital lobe, which may be associated with the pathology found in the occipital lobe (Thientunyakit et al., 2021) and abnormal cerebral blood flow in the occipital lobe (Alexopoulos et al., 2012).In recent years, there have been reports suggesting abnormal interhemispheric connectivity in patients with Alzheimer's disease (AD). The main impact of this finding on the assessment of AD patients is likely to be more selective at the preliminary screening stage, and it may be possible to monitor their disease progression using noninvasive EEG methods (Vecchio et al., 2015, 2018). Our results are consistent with these observations. Abnormal specialization is mainly caused by reduced cross‐hemispheric connectivity with the temporal lobe. The temporal lobe, together with its subcortical area, the hippocampus, is crucial for memory. In addition, the occipital lobe is a brain area involved in multiple cognitive processes. A circuit that modulates episodic memory has been observed between the occipital lobe and hippocampus (Hebscher et al., 2021). Episodic memory can be impaired when a circuit is disconnected (Tambini & D'Esposito, 2020). It is suggested that future studies should consider the left occipital lobe as a potential area to focus on in order to promote the improvement of AD symptoms.

Corpus callosum mainly emphasizes structural coordination, which requires the support of diffusion tensor imaging (DTI). We do not have DTI results, but DTI focuses on white matter results, and this study mainly emphasizes functional coordination and specialization. In our study, we observed a significant decrease in cooperation between the bilateral precuneus and prefrontal cortex. The precuneus, a region within the default mode network (DMN) (Raichle, 2015), is known to play a crucial role in cognitive processes according to previous research (Cavanna & Trimble, 2006). Other studies have suggested that transcranial magnetic stimulation (TMS) could be an effective method for enhancing therapy in AD patients. In fact, the precuneus (PC) has been identified as a target for TMS therapy based on current guidelines (Lefaucheur et al., 2020). A recent notable study reported that rTMS stimulation of the PC can serve as a treatment approach for AD. The stimulation was found to improve specific episodic memory in patients and modulate connectivity between the parietal, frontal, and temporal regions. These findings provide initial evidence for the effectiveness of noninvasive stimulation targeting the PC in improving cognitive impairments in AD (Koch & Spampinato, 2022). Our findings contribute to the existing evidence supporting the precuneus as a potential target for TMS treatment in individuals with AD. We also observed that cooperation in the bilateral prefrontal cortex was abnormal. The prefrontal cortex is vital for many cognitive functions and is located in many intrinsic functional networks, such as the executive control network (ECN) and parietal–frontal network (Dajani & Uddin, 2015), which are dysfunctional in AD progression (Chong et al., 2017; Dai et al., 2019; Lehmann et al., 2013). The original functions of these networks play a vital role in the preservation and manipulation of information in the working memory, solving problems based on rules, as well as making decisions in the context of behavior aimed at achieving goals (Chand & Dhamala, 2016). Our results suggest the abnormal function of intrinsic functional networks, which is in line with previous studies that showed that AD is a network disease (Myers et al., 2014). For example, the Salience Network (SN) not only plays a specialized role in several higher‐order cognitive functions, but also acts as a mediator in the triple‐network model, providing a more sensitive biomarker for overall cognitive performance in AD. Our research focuses on the functional integration and specialization of the brain, and therefore, the abnormality of the bilateral SN in the brain may lead to impaired specialization. Since the severity of the disease varies among the patients participating in the study, further studies with a larger sample size may be needed (Zhang et al., 2022).

Taken together, our results showing a correlation between abnormal prefrontal cooperation and cognitive performance were reasonable. The abnormal cooperation suggests the disruption of intrinsic functional networks, whose function is important in memory or other multiple cognitive processes. Severe network disruption may indicate poor cognitive performance. The machine learning process based on SVM also suggested that disruption of the DMN and ECN networks may be a prominent alteration of the disease, which is in line with the triple‐network theory of dementia observed in previous studies (Li et al., 2019). Although the SVM results did not show better reliability and validity than traditional methods, we employed SVM to demonstrate that there are indeed differences between AD and HC in terms of the AI and CFH indices, not to prove that these indices can replace cognitive scales such as MMSE and MoCA in the diagnosis of AD.

There were certain limitations in our study. First, the selection of participants was based on the NINCDS‐ADRDA criteria, resulting in a sample consisting of patients clinically diagnosed with probable AD. The absence of biomarkers might have introduced some degree of bias. Second, the sample size of our study was too small to classify AD according to light, medium and heavy subgroups, so further sample expansion is needed for analysis. Third, fMRI relied on blood oxygenation imaging, which was slower than directly measuring brain electrical activity. It had high spatial resolution, but its temporal resolution was not as high as that of EEG, therefore it could not explore neural phase information. Lastly, our study included only one cohort, and further validation in other cohorts, such as the Alzheimer's Disease Neuroimaging Initiative (ADNI) database, would be beneficial.

## 5 CONCLUSION

Disruption of cerebral specialization and interhemispheric cooperation in AD could potentially underlie cognitive decline as the disease progresses. This dysfunction might serve as a basis for identifying novel biomarkers in the future.

## AUTHOR CONTRIBUTIONS

**Yue Wu**: Writing—original draft; formal analysis; methodology; software; visualization; investigation; data curation. **Manman Gao**: Writing—original draft; writing—review and editing; formal analysis; visualization; investigation; data curation. **Lingling Lv**: Investigation; data curation. **Yibing Yan**: Investigation; data curation. **Liying Gao**: Investigation; validation. **Zhi Geng**: Investigation; data curation. **Shanshan Zhou**: Investigation; data curation. **Wanqiu Zhu**: Investigation; data curation. **Yongqiang Yu**: Investigation; data curation. **Yanghua Tian**: Supervision; funding acquisition. **Gong‐Jun Ji**: Methodology; software. **Panpan Hu**: Funding acquisition; supervision; resources; conceptualization. **Xingqi Wu**: Funding acquisition; supervision; resources. **Kai Wang**: Conceptualization; funding acquisition; supervision; resources.

## CONFLICT OF INTEREST STATEMENT

There is no interest of conflict.

### PATIENT CONSENT STATEMENT

The patients/participants provided their written informed consent to participate in this study.

### PERMISSION TO REPRODUCE MATERIAL FROM OTHER SOURCES

The figure in the manuscript was not from other sources.

### PEER REVIEW

The peer review history for this article is available at https://publons.com/publon/10.1002/brb3.3550

## Supporting information

Figure S1: Scatter diagram of AI showing differences between groups AI of the left middle occipital lobe was increased in the AD group.
Figure S2: FC of left middle occipital lobe with right temporal lobe. (A) Based on the seed point of the left middle occipital lobe that showed AI differences between groups, FC of left middle occipital lobe with right temporal lobe was decreased in the AD group. (B) Scatter diagram of the FC between left middle occipital lobe and right temporal lobe. The FC between left middle occipital lobe and right temporal lobe was decreased in the AD group.
Figure S3: Scatter diagram of CFH showing differences between groups. (A) CFH of the left precuneus was decreased in the AD group. (B) CFH of the right precuneus was decreased in the AD group. (C) CFH of the left prefrontal cortex was decreased in the AD group. (D) CFH of the right prefrontal cortex was decreased in the AD group.
Table S1 Brain regions of FC based on the left middle occipital lobe differences between groups.

## Data Availability

The datasets used and/or analyzed during the current study are available from the corresponding author on reasonable request.

## References

[brb33550-bib-0001] Alexopoulos, P. , Sorg, C. , Förschler, A. , Grimmer, T. , Skokou, M. , Wohlschläger, A. , Perneczky, R. , Zimmer, C. , Kurz, A. , & Preibisch, C. (2012). Perfusion abnormalities in mild cognitive impairment and mild dementia in Alzheimer's disease measured by pulsed arterial spin labeling MRI. European Archives of Psychiatry and Clinical Neuroscience, 262(1), 69–77. 10.1007/s00406-011-0226-2 21786091

[brb33550-bib-0002] Arvanitakis, Z. , Shah, R. C. , & Bennett, D. A. (2019). Diagnosis and management of dementia: Review. Jama, 322(16), 1589–1599. 10.1001/jama.2019.4782 31638686 PMC7462122

[brb33550-bib-0003] Cavanna, A. E. , & Trimble, M. R. (2006). The precuneus: A review of its functional anatomy and behavioural correlates. Brain: A Journal of Neurology, 129(Pt 3), 564–583. 10.1093/brain/awl004 16399806

[brb33550-bib-0004] Chand, G. B. , & Dhamala, M. (2016). Interactions among the brain default‐mode, salience, and central‐executive networks during perceptual decision‐making of moving dots. Brain Connectivity, 6(3), 249–254. 10.1089/brain.2015.0379 26694702

[brb33550-bib-0005] Chang, C.‐C. , & Lin, C.‐J. (2011). LIBSVM: A library for support vector machines. ACM Transactions on Intelligent Systems and Technology (TIST), 2(3), 1–27. 10.1145/1961189.1961199

[brb33550-bib-0006] Chong, J. S. X. , Liu, S. , Loke, Y. M. , Hilal, S. , Ikram, M. K. , Xu, X. , Tan, B. Y. , Venketasubramanian, N. , Chen, C. L.‐H. , & Zhou, J. (2017). Influence of cerebrovascular disease on brain networks in prodromal and clinical Alzheimer's disease. Brain: A Journal of Neurology, 140(11), 3012–3022. 10.1093/brain/awx224 29053778 PMC5841199

[brb33550-bib-0007] Dai, Z. , Lin, Q. , Li, T. , Wang, X. , Yuan, H. , Yu, X. , He, Y. , & Wang, H. (2019). Disrupted structural and functional brain networks in Alzheimer's disease. Neurobiology of Aging, 75, 71–82. 10.1016/j.neurobiolaging.2018.11.005 30553155

[brb33550-bib-0008] Dajani, D. R. , & Uddin, L. Q. (2015). Demystifying cognitive flexibility: Implications for clinical and developmental neuroscience. Trends in Neurosciences, 38(9), 571–578. 10.1016/j.tins.2015.07.003 26343956 PMC5414037

[brb33550-bib-0009] Davis, S. W. , & Cabeza, R. (2015). Cross‐hemispheric collaboration and segregation associated with task difficulty as revealed by structural and functional connectivity. The Journal of Neuroscience: The Official Journal of the Society for Neuroscience, 35(21), 8191–8200. 10.1523/JNEUROSCI.0464-15.2015 26019335 PMC4444541

[brb33550-bib-0010] Frings, L. , Hellwig, S. , Spehl, T. S. , Bormann, T. , Buchert, R. , Vach, W. , Minkova, L. , Heimbach, B. , Klöppel, S. , & Meyer, P. T. (2015). Asymmetries of amyloid‐β burden and neuronal dysfunction are positively correlated in Alzheimer's disease. Brain: A Journal of Neurology, 138(Pt 10), 3089–3099. 10.1093/brain/awv229 26280595

[brb33550-bib-0011] Gazzaniga, M. S. (2000). Cerebral specialization and interhemispheric communication: Does the corpus callosum enable the human condition? Brain: A Journal of Neurology, 123(Pt 7), 1293–1326. 10.1093/brain/123.7.1293 10869045

[brb33550-bib-0012] Ge, X. , Zhang, D. , Qiao, Y. , Zhang, J. , Xu, J. , & Zheng, Y. (2021). Association of Tau pathology with clinical symptoms in the subfields of hippocampal formation. Frontiers in Aging Neuroscience, 13, 672077. 10.3389/fnagi.2021.672077 34335226 PMC8317580

[brb33550-bib-0013] Geschwind, N. , & Levitsky, W. (1968). Human brain: Left‐right asymmetries in temporal speech region. Science (New York, N.Y.), 161(3837), 186–187. 10.1126/science.161.3837.186 5657070

[brb33550-bib-0014] Golby, A. , Silverberg, G. , Race, E. , Gabrieli, S. , O'Shea, J. , Knierim, K. , Stebbins, G. , & Gabrieli, J. (2005). Memory encoding in Alzheimer's disease: An fMRI study of explicit and implicit memory. Brain: A Journal of Neurology, 128(Pt 4), 773–787. 10.1093/brain/awh400 15705615

[brb33550-bib-0015] Golby, A. J. , Poldrack, R. A. , Brewer, J. B. , Spencer, D. , Desmond, J. E. , Aron, A. P. , & Gabrieli, J. D. (2001). Material‐specific lateralization in the medial temporal lobe and prefrontal cortex during memory encoding. Brain: A Journal of Neurology, 124(Pt 9), 1841–1854. 10.1093/brain/124.9.1841 11522586

[brb33550-bib-0016] Hebscher, M. , Kragel, J. E. , Kahnt, T. , & Voss, J. L. (2021). Enhanced reinstatement of naturalistic event memories due to hippocampal‐network‐targeted stimulation. Current Biology: CB, 31(7), 1428–1437.e5. 10.1016/j.cub.2021.01.027 33545044 PMC8044012

[brb33550-bib-0017] jr Jack, C. R., ., Bennett, D. A. , Blennow, K. , Carrillo, M. C. , Dunn, B. , Haeberlein, S. B. , Holtzman, D. M. , Jagust, W. , Jessen, F. , Karlawish, J. , Liu, E. , Molinuevo, J. L. , Montine, T. , Phelps, C. , Rankin, K. P. , Rowe, C. C. , Scheltens, P. , Siemers, E. , Snyder, H. M. , … Contributors . (2018). NIA‐AA Research Framework: Toward a biological definition of Alzheimer's disease. Alzheimer's & dementia: The journal of the Alzheimer's. Association, 14(4), 535–562. 10.1016/j.jalz.2018.02.018 PMC5958625 29653606

[brb33550-bib-0018] Janota, I. , & Mountjoy, C. Q. (1988). Asymmetry of pathology in Alzheimer's disease. Journal of Neurology, Neurosurgery, and Psychiatry, 51(7), 1011–1012. 10.1136/jnnp.51.7.1011-c PMC1033227 3204396

[brb33550-bib-0019] Jo, H. J. , Saad, Z. S. , Gotts, S. J. , Martin, A. , & Cox, R. W. (2012). Quantifying agreement between anatomical and functional interhemispheric correspondences in the resting brain. PLoS ONE, 7(11), e48847. 10.1371/journal.pone.0048847 23144995 PMC3493608

[brb33550-bib-0020] Karolis, V. R. , Corbetta, M. , & Thiebaut De Schotten, M. (2019). The architecture of functional lateralisation and its relationship to callosal connectivity in the human brain. Nature Communications, 10(1), 1417. 10.1038/s41467-019-09344-1 PMC6441088 30926845

[brb33550-bib-0021] Kelley, W. M. , Miezin, F. M. , Mcdermott, K. B. , Buckner, R. L. , Raichle, M. E. , Cohen, N. J. , Ollinger, J. M. , Akbudak, E. , Conturo, T. E. , Snyder, A. Z. , & Petersen, S. E. (1998). Hemispheric specialization in human dorsal frontal cortex and medial temporal lobe for verbal and nonverbal memory encoding. Neuron, 20(5), 927–936. 10.1016/S0896-6273(00)80474-2 9620697

[brb33550-bib-0022] Koch, G. , & Spampinato, D. (2022). Alzheimer disease and neuroplasticity. Handbook of Clinical Neurology, 184, 473–479. 10.1016/B978-0-12-819410-2.00027-8 35034755

[brb33550-bib-0023] Lefaucheur, J.‐P. , Aleman, A. , Baeken, C. , Benninger, D. H. , Brunelin, J. , Di Lazzaro, V. , Filipović, S. R. , Grefkes, C. , Hasan, A. , Hummel, F. C. , Jääskeläinen, S. K. , Langguth, B. , Leocani, L. , Londero, A. , Nardone, R. , Nguyen, J.‐P. , Nyffeler, T. , Oliveira‐Maia, A. J. , Oliviero, A. , … Ziemann, U. (2020). Evidence‐based guidelines on the therapeutic use of repetitive transcranial magnetic stimulation (rTMS): An update (2014‐2018). Clinical Neurophysiology: Official Journal of the International Federation of Clinical Neurophysiology, 131(2), 474–528. 10.1016/j.clinph.2019.11.002 31901449

[brb33550-bib-0024] Lehmann, M. , Ghosh, P. M. , Madison, C. , Laforce, R. , Corbetta‐Rastelli, C. , Weiner, M. W. , Greicius, M. D. , Seeley, W. W. , Gorno‐Tempini, M. L. , Rosen, H. J. , Miller, B. L. , Jagust, W. J. , & Rabinovici, G. D. (2013). Diverging patterns of amyloid deposition and hypometabolism in clinical variants of probable Alzheimer's disease. Brain: A Journal of Neurology, 136(Pt 3), 844–858. 10.1093/brain/aws327 23358601 PMC3580269

[brb33550-bib-0025] Li, C. , Li, Y. , Zheng, L. , Zhu, X. , Shao, B. , Fan, G. , Liu, T. , & Wang, J. (2019). Abnormal brain network connectivity in a triple‐network model of Alzheimer's disease. Journal of Alzheimer's Disease: JAD, 69(1), 237–252. 10.3233/JAD-181097 30958354

[brb33550-bib-0026] Li, J. , Yuan, Y. , Wang, M. , Zhang, J. , Zhang, L. , Jiang, S. , Wang, X. , Ding, J. , & Zhang, K. (2018). Decreased interhemispheric homotopic connectivity in Parkinson's disease patients with freezing of gait: A resting state fMRI study. Parkinsonism & Related Disorders, 52, 30–36. 10.1016/j.parkreldis.2018.03.015 29602542

[brb33550-bib-0027] Luders, E. , Gaser, C. , Jancke, L. , & Schlaug, G. (2004). A voxel‐based approach to gray matter asymmetries. Neuroimage, 22(2), 656–664. 10.1016/j.neuroimage.2004.01.032 15193594

[brb33550-bib-0028] Madsen, S. K. , Ho, A. J. , Hua, X. , Saharan, P. S. , Toga, A. W. , Jack, C. R. , Weiner, M. W. , & Thompson, P. M. (2010). 3D maps localize caudate nucleus atrophy in 400 Alzheimer's disease, mild cognitive impairment, and healthy elderly subjects. Neurobiology of Aging, 31(8), 1312–1325. 10.1016/j.neurobiolaging.2010.05.002 20538376 PMC2903198

[brb33550-bib-0029] Malpetti, M. , Kievit, R. A. , Passamonti, L. , Jones, P. S. , Tsvetanov, K. A. , Rittman, T. , Mak, E. , Nicastro, N. , Bevan‐Jones, W. R. , Su, L. , Hong, Y. T. , Fryer, T. D. , Aigbirhio, F. I. , O'brien, J. T. , & Rowe, J. B. (2020). Microglial activation and tau burden predict cognitive decline in Alzheimer's disease. Brain: A Journal of Neurology, 143(5), 1588–1602. 10.1093/brain/awaa088 32380523 PMC7241955

[brb33550-bib-0030] Mcdonough, I. M. , Festini, S. B. , & Wood, M. M. (2020). Risk for Alzheimer's disease: A review of long‐term episodic memory encoding and retrieval fMRI studies. Ageing Research Reviews, 62, 101133. 10.1016/j.arr.2020.101133 32717407

[brb33550-bib-0031] Mckhann, G. , Drachman, D. , Folstein, M. , Katzman, R. , Price, D. , & Stadlan, E. M. (1984). Clinical diagnosis of Alzheimer's disease: Report of the NINCDS‐ADRDA work group under the auspices of department of health and human services task force on Alzheimer's disease. Neurology, 34(7), 939–944. 10.1212/WNL.34.7.939 6610841

[brb33550-bib-0032] Minkova, L. , Habich, A. , Peter, J. , Kaller, C. P. , Eickhoff, S. B. , & Klöppel, S. (2017). Gray matter asymmetries in aging and neurodegeneration: A review and meta‐analysis. Human Brain Mapping, 38(12), 5890–5904. 10.1002/hbm.23772 28856766 PMC6866813

[brb33550-bib-0033] Mueller, S. , Wang, D. , Pan, R. , Holt, D. J. , & Liu, H. (2015). Abnormalities in hemispheric specialization of caudate nucleus connectivity in schizophrenia. JAMA Psychiatry, 72(6), 552–560. 10.1001/jamapsychiatry.2014.3176 25830688 PMC4630217

[brb33550-bib-0034] Myers, N. , Pasquini, L. , Göttler, J. , Grimmer, T. , Koch, K. , Ortner, M. , Neitzel, J. , Mühlau, M. , Förster, S. , Kurz, A. , Förstl, H. , Zimmer, C. , Wohlschläger, A. M. , Riedl, V. , Drzezga, A. , & Sorg, C. (2014). Within‐patient correspondence of amyloid‐β and intrinsic network connectivity in Alzheimer's disease. Brain: A Journal of Neurology, 137(Pt 7), 2052–2064. 10.1093/brain/awu103 24771519 PMC4065018

[brb33550-bib-0035] Raichle, M. E. (2015). The brain's default mode network. Annual Review of Neuroscience, 38, 433–447. 10.1146/annurev-neuro-071013-014030 25938726

[brb33550-bib-0036] Roe, J. M. , Vidal‐Piñeiro, D. , Sørensen, Ø. , Brandmaier, A. M. , Düzel, S. , Gonzalez, H. A. , Kievit, R. A. , Knights, E. , Kühn, S. , Lindenberger, U. , Mowinckel, A. M. , Nyberg, L. , Park, D. C. , Pudas, S. , Rundle, M. M. , Walhovd, K. B. , Fjell, A. M. , Westerhausen, R. , Masters, C. L. , … Vacher, M. (2021). Asymmetric thinning of the cerebral cortex across the adult lifespan is accelerated in Alzheimer's disease. Nature Communications, 12(1), 721. 10.1038/s41467-021-21057-y PMC7851164 33526780

[brb33550-bib-0037] Rogers, L. J. , Zucca, P. , & Vallortigara, G. (2004). Advantages of having a lateralized brain. Proceedings. Biological Sciences, 271 Suppl 271, (Suppl 6), S420–S422. 10.1098/rsbl.2004.0200 15801592 PMC1810119

[brb33550-bib-0038] Spagna, A. , Kim, T. H. , Wu, T. , & Fan, J. (2020). Right hemisphere superiority for executive control of attention. Cortex; A Journal Devoted to the Study of the Nervous System and Behavior, 122, 263–276. 10.1016/j.cortex.2018.12.012 30661735

[brb33550-bib-0039] Sun, J. , Gao, X. , Hua, Q. , Du, R. , Liu, P. , Liu, T. , Yang, J. , Qiu, B. , Ji, G.‐J. , Hu, P. , & Wang, K. (2022). Brain functional specialization and cooperation in Parkinson's disease. Brain Imaging and Behavior, 16(2), 565–573. 10.1007/s11682-021-00526-4 34427879

[brb33550-bib-0040] Tambini, A. , & D'esposito, M. (2020). Causal contribution of Awake post‐encoding processes to episodic memory consolidation. Current Biology: CB, 30(18), 3533–3543.e7. 10.1016/j.cub.2020.06.063 32735812 PMC7511431

[brb33550-bib-0041] Thientunyakit, T. , Thongpraparn, T. , Sethanandha, C. , Yamada, T. , Kimura, Y. , Muangpaisan, W. , & Ishii, K. (2021). Relationship between F‐18 florbetapir uptake in occipital lobe and neurocognitive performance in Alzheimer's disease. Japanese Journal of Radiology, 39(10), 984–993. 10.1007/s11604-021-01132-6 34019227

[brb33550-bib-0042] Vecchio, F. , Miraglia, F. , Curcio, G. , Altavilla, R. , Scrascia, F. , Giambattistelli, F. , Quattrocchi, C. C. , Bramanti, P. , Vernieri, F. , & Rossini, P. M. (2015). Cortical brain connectivity evaluated by graph theory in dementia: A correlation study between functional and structural data. Journal of Alzheimer's Disease: JAD, 45(3), 745–756. 10.3233/JAD-142484 25613102

[brb33550-bib-0043] Vecchio, F. , Miraglia, F. , Quaranta, D. , Lacidogna, G. , Marra, C. , & Rossini, P. M. (2018). Learning processes and brain connectivity in A cognitive‐motor task in neurodegeneration: Evidence from EEG network analysis. Journal of Alzheimer's Disease: JAD, 66(2), 471–481. 10.3233/JAD-180342 30282357

[brb33550-bib-0044] Wachinger, C. , Nho, K. , Saykin, A. J. , Reuter, M. , & Rieckmann, A. (2018). A longitudinal imaging genetics study of neuroanatomical asymmetry in Alzheimer's disease. Biological Psychiatry, 84(7), 522–530. 10.1016/j.biopsych.2018.04.017 29885764 PMC6123250

[brb33550-bib-0045] Walker, J. M. , Fudym, Y. , Farrell, K. , Iida, M. A. , Bieniek, K. F. , Seshadri, S. , White, C. L. , Crary, J. F. , & Richardson, T. E. (2021). Asymmetry of Hippocampal Tau pathology in primary age‐related tauopathy and Alzheimer disease. Journal of Neuropathology and Experimental Neurology, 80(5), 436–445. 10.1093/jnen/nlab032 33860327 PMC8054137

[brb33550-bib-0046] Wang, D. , Buckner, R. L. , & Liu, H. (2014). Functional specialization in the human brain estimated by intrinsic hemispheric interaction. The Journal of Neuroscience: The Official Journal of the Society for Neuroscience, 34(37), 12341–12352. 10.1523/JNEUROSCI.0787-14.2014 25209275 PMC4160771

[brb33550-bib-0047] Whitwell, J. L. , Graff‐Radford, J. , Tosakulwong, N. , Weigand, S. D. , Machulda, M. M. , Senjem, M. L. , Spychalla, A. J. , Vemuri, P. , Jones, D. T. , Drubach, D. A. , Knopman, D. S. , Boeve, B. F. , Ertekin‐Taner, N. , Petersen, R. C. , Lowe, V. J. , jr Jack, C. R., , & Josephs, K. A. (2018). Imaging correlations of tau, amyloid, metabolism, and atrophy in typical and atypical Alzheimer's disease. Alzheimer's & Dementia: The Journal of the Alzheimer's Association, 14(8), 1005–1014. 10.1016/j.jalz.2018.02.020 PMC6097955 29605222

[brb33550-bib-0048] Wu, X. , Wu, Y. , Geng, Z. , Zhou, S. , Wei, L. , Ji, G.‐J. , Tian, Y. , & Wang, K. (2020). Asymmetric differences in the gray matter volume and functional connections of the amygdala are associated with clinical manifestations of Alzheimer's disease. Frontiers in Neuroscience, 14, 602. 10.3389/fnins.2020.00602 32670008 PMC7332559

[brb33550-bib-0049] Yan, C. G. , Wang, X. D. , Zuo, X. N. , & Zang, Y. F. (2016). DPABI: Data processing & analysis for (resting‐state) brain imaging. Neuroinformatics, 14(3), 339–351. 10.1007/s12021-016-9299-4 27075850

[brb33550-bib-0050] Zhang, M. , Guan, Z. , Zhang, Y. , Sun, W. , Li, W. , Hu, J. , Li, B. , Ye, G. , Meng, H. , Huang, X. , Lin, X. , Wang, J. , Liu, J. , Li, B. , & Li, Y. (2022). Disrupted coupling between salience network segregation and glucose metabolism is associated with cognitive decline in Alzheimer's disease—A simultaneous resting‐state FDG‐PET/fMRI study. NeuroImage. Clinical, 34, 102977. 10.1016/j.nicl.2022.102977 35259618 PMC8904621

[brb33550-bib-0051] Zuo, X.‐N. , Kelly, C. , Di Martino, A. , Mennes, M. , Margulies, D. S. , Bangaru, S. , Grzadzinski, R. , Evans, A. C. , Zang, Y.‐F. , Castellanos, F. X. , & Milham, M. P. (2010). Growing together and growing apart: Regional and sex differences in the lifespan developmental trajectories of functional homotopy. The Journal of Neuroscience: The Official Journal of the Society for Neuroscience, 30(45), 15034–15043. 10.1523/JNEUROSCI.2612-10.2010 21068309 PMC2997358

