# Supplementary material for: Brain functional specialization and cooperation in Alzheimer's disease
Source: Brain Behav. 2024 Jun 6;14(6):e3550. doi: 10.1002/brb3.3550 (PMC11154812; doi:10.1002/brb3.3550)
Supplement: Supplementary file 1 — Figure S1: Scatter diagram of AI showing differences between groups AI of the left middle occipital lobe was increased in the AD group. Figure S2: FC of left middle occipital lobe with right temporal lobe. (A) Based on the seed point of the left middle occipital lobe that showed AI differences between groups, FC of left middle occipital lobe with right temporal lobe was decreased in the AD group. (B) Scatter diagram of the FC between left middle occipital lobe and right temporal lobe. The FC between left middle occipital lobe and right temporal lobe was decreased in the AD group. Figure S3: Scatter diagram of CFH showing differences between groups. (A) CFH of the left precuneus was decreased in the AD group. (B) CFH of the right precuneus was decreased in the AD group. (C) CFH of the left prefrontal cortex was decreased in the AD group. (D) CFH of the right prefrontal cortex was decreased in the AD group. Table S1 Brain regions of FC based on the left middle occipital lobe differences between groups. [file BRB3-14-e3550-s001.docx]

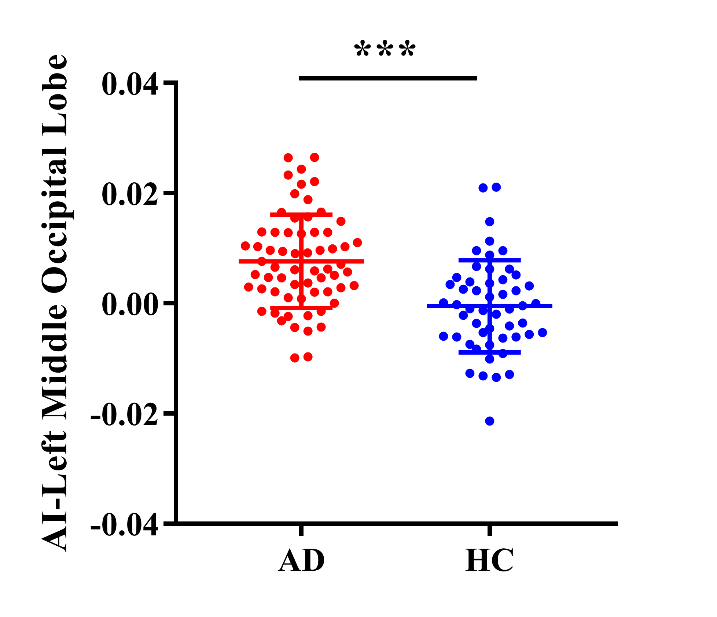


Figure S1: Scatter Diagram of AI showing differences between groups

AI of the left middle occipital lobe was increased in the AD group.


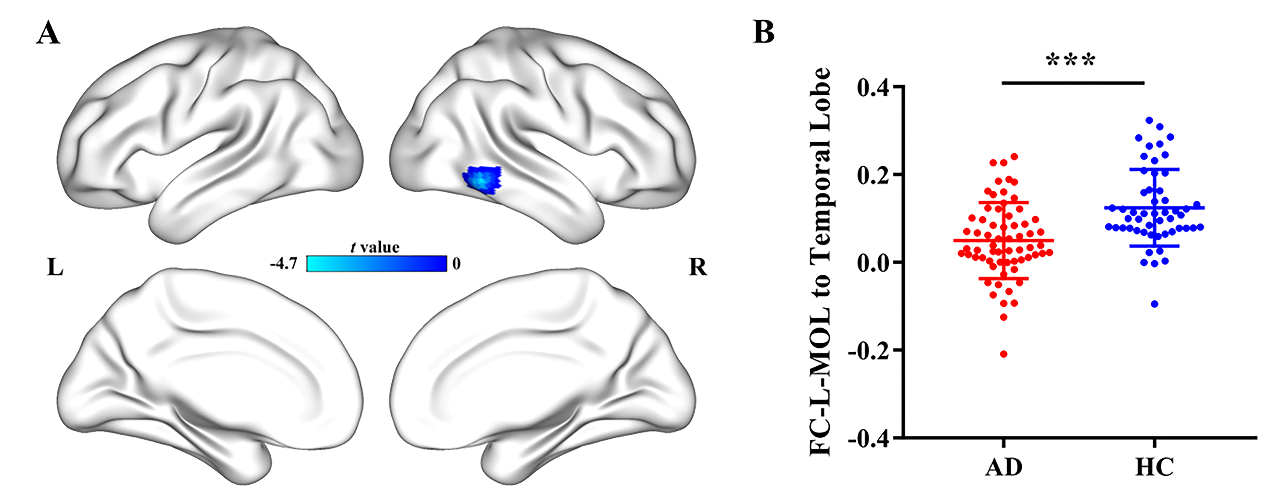


Figure S2: FC of Left Middle Occipital Lobe with Right Temporal Lobe

A: Based on the seed point of the left middle occipital lobe that showed AI differences between groups, FC of left middle occipital lobe with right temporal lobe was decreased in the AD group. B: Scatter diagram of the FC between left middle occipital lobe and right temporal lobe. The FC between left middle occipital lobe and right temporal lobe was decreased in the AD group.

Table S1 Brain Regions of FC Based on the Left Middle Occipital Lobe differences between groups

| Brain Regions | Peak MNI coordinates | | | Voxels | *t* | *p _GRF_*_-corr_ |
| --- | --- | --- | --- | --- | --- | --- |
|  | x | y | z |  |  |  |
| R Temporal Lobe | 54 | -48 | -9 | 23 | 4.70 | <.06^+^ |

+: Marginally decreased FC of left middle occipital lobe with right temporal lobe was observed based on Gaussian Random Field (GRF) method, with voxel level set at *p* < .0001 and cluster level set at *p* < .06.


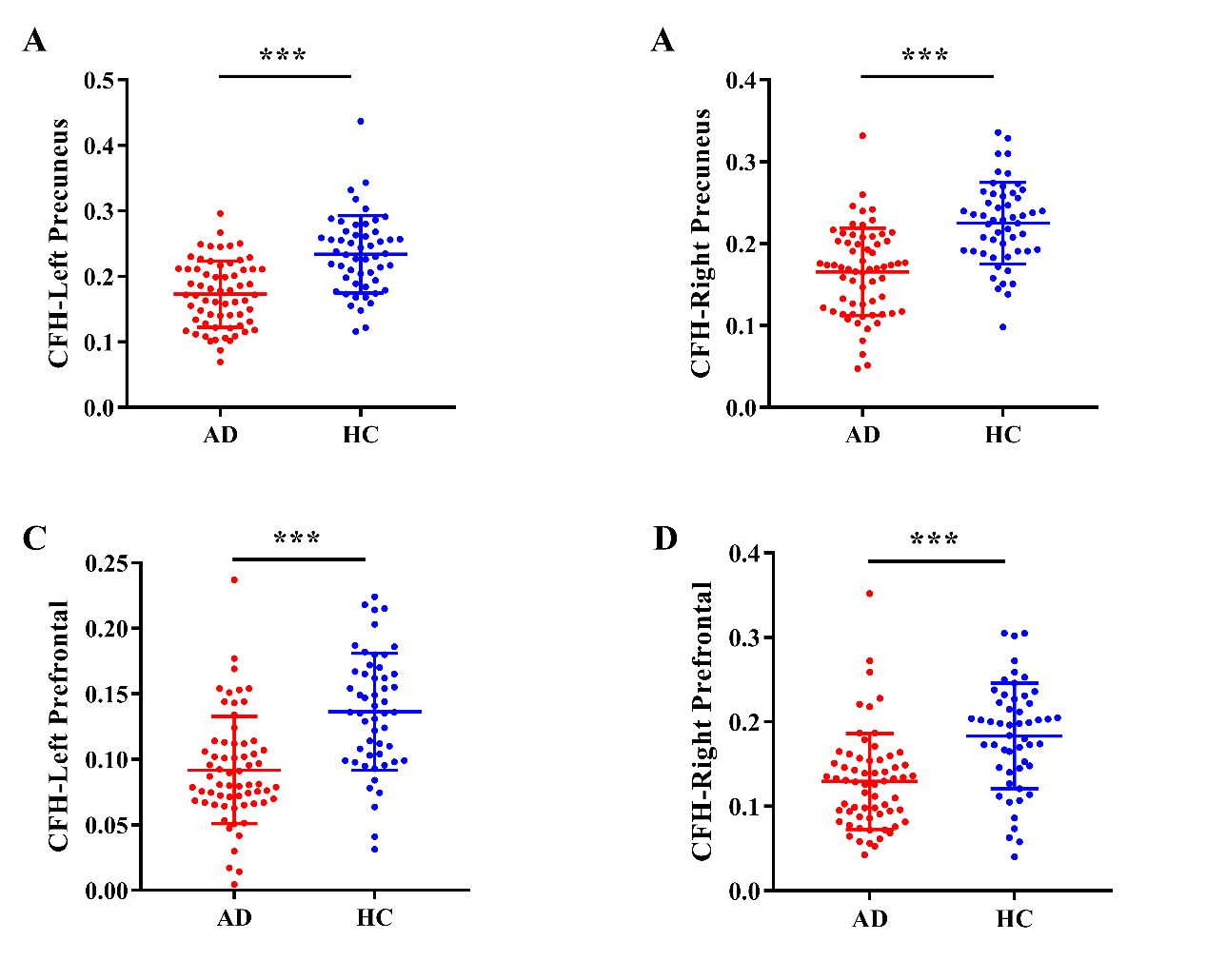


Figure S3: Scatter Diagram of CFH showing differences between groups

A: CFH of the left precuneus was decreased in the AD group. B: CFH of the right precuneus was decreased in the AD group. C: CFH of the left prefrontal cortex was decreased in the AD group. D: CFH of the right prefrontal cortex was decreased in the AD group.
